# Novel Insights into the Sinoatrial Node in Single-Cell RNA Sequencing: From Developmental Biology to Physiological Function

**DOI:** 10.3390/jcdd9110402

**Published:** 2022-11-18

**Authors:** Wei Fan, Chao Yang, Xiaojie Hou, Juyi Wan, Bin Liao

**Affiliations:** 1 Department of Cardiovascular Surgery, The Affiliated Hospital of Southwest Medical University, Luzhou 646000, China; 2 Metabolic Vascular Diseases Key Laboratory of Sichuan Province, Luzhou 646000, China; 3 Key Laboratory of Medical Electrophysiology, Ministry of Education & Medical Electrophysiological Key Laboratory of Sichuan Province, (Collaborative Innovation Center for Prevention of Cardiovascular Diseases), Institute of Cardiovascular Research, Southwest Medical University, Luzhou 646000, China; 4 Department of Cardiac Surgery, Beijing Anzhen Hospital, Capital Medical University, Beijing 100069, China

**Keywords:** sinoatrial node, single-cell RNA sequencing, transcription factors, signaling pathways, molecular regulation

## Abstract

Normal cardiac automaticity is dependent on the pacemaker cells of the sinoatrial node (SAN). Insufficient cardiac pacemaking leads to the development of sick sinus syndrome (SSS). Since currently available pharmaceutical drugs and implantable pacemakers are only partially effective in managing SSS, there is a critical need for developing targeted mechanism-based therapies to treat SSS. SAN-like pacemaker cells (SANLPCs) are difficult to regenerate in vivo or in vitro because the genes and signaling pathways that regulate SAN development and function have not been fully elucidated. The development of more effective treatments for SSS, including biological pacemakers, requires further understanding of these genes and signaling pathways. Compared with genetic models and bulk RNA sequencing, single-cell RNA sequencing (scRNA-seq) technology promises to advance our understanding of cellular phenotype heterogeneity and molecular regulation during SAN development. This review outlines the key transcriptional networks that control the structure, development, and function of the SAN, with particular attention to SAN markers and signaling pathways detected via scRNA-seq. This review offers insights into the process and transcriptional network of SAN morphogenesis at a single-cell level and discusses current challenges and potential future directions for generating SANLPCs for biological pacemakers.

## 1. Introduction

The sinoatrial node (SAN) is a small population of cardiomyocytes that spontaneously fire to trigger each heartbeat. Abnormal SAN formation or function can lead to sick sinus syndrome (SSS), including sinus bradycardia, sinoatrial arrest/block, bradycardia–tachycardia syndrome, and sudden death [1]. Currently, no drugs for long-term use are effective for SSS treatment [2]. Development of more effective treatments for SSS, such as biological pacemakers, requires a comprehensive understanding of the genes and signaling pathways involved in the regulation of SAN development and function. Since the discovery of the SAN more than a century ago, studies have uncovered its intricate molecular structure and unique ion channels expressed within its myocytes. However, the molecular and cellular features that influence the pivotal functions of the SAN in the heart have not been fully elucidated [2].

The SAN is a subtle biological system that comprises multiple subdomains and cell types with distinct functions, including pacemaker cells (PCs) and several non-PCs [3]. Some previous studies that used genetically deficient mice (*TBX3, TBX5, TBX18, SHOX2, NKX2-5,* and *PITX2*) provided initial insights into SAN development and function [4,5,6,7,8]. Unfortunately, these studies are often limited by the small size and heterogeneous organization of the SAN, providing insufficient knowledge on gene regulation of the SAN [9]. Bulk RNA sequencing (RNA-seq) provides transcriptome data for the study of the SAN, further advancing SAN research. However, given the lack of specific molecular markers and the small size of the SAN, transcriptome data are often distorted and affected by other cells [10]. Despite significant progress in SAN research, our understanding of its cellular features, differentiation trajectories, and regulatory mechanisms is incomplete. Some obstacles in investigating the molecular features of the SAN include (1) the low total number of PCs in the heart, (2) the complex three-dimensional anatomy of the SAN, (3) difficulties in separating PCs from the surrounding myocardium, (4) lack of specific molecular markers, and (5) significant cell heterogeneity in SAN tissues [10,11].

The application of single-cell RNA sequencing (scRNA-seq) technology has enabled the investigation of gene regulation, cell diversification and temporality, cellular heterogeneity, and developmental temporality from a global and unbiased perspective with unprecedented resolution [12]. The scRNA-seq technology can overcome several obstacles in SAN research, including low cell numbers, complex and variable structures, distorted transcriptional profiles due to cell heterogeneity, and nonconductive cell contamination [10]. In the present study, we used scRNA-seq to explore the spatial and temporal changes that occur during heart development in mice, identify SAN cell populations, and clarify their cellular heterogeneity and precise anatomical location [13,14].

An improved understanding of SAN at the cellular level will facilitate the development of novel and more effective therapeutic strategies for SSS. Herein, we review recent advances in single-cell transcriptomic analysis to characterize the structure, core transcription factors (TFs), novel specific molecular markers, signaling pathways, and physiological properties of the SAN.

## 2. SAN Structure

**Figure 1:**
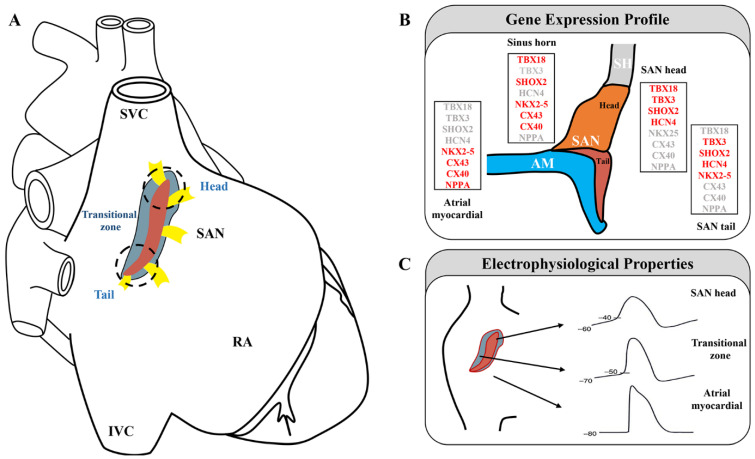
Sinoatrial node subdomains are divided based on anatomical and genetic criteria. (**A**) The sinoatrial node is displayed on a schematic diagram of the heart (red, central SAN region; blue, transitional cells; yellow, sinoatrial conduction pathways; white dashed line, SAN head; black dashed line, SAN tail). Adapted with permission from [26,30], Copyright (2005, 2017). (**B**) Gene expression profile in the SAN region (expressed genes are indicated in red). Adapted with permission from [7], Copyright (2009). (**C**) Samples of action potentials (APs) are recorded from the central sinoatrial node to the periphery of the node. Adapted with permission from [22], Copyright (2016).

The human SAN is a compact, slightly elongated, banana-shaped structure located at the junction of the lateral superior vena cava (SVC) and right atrium (RA). Functional and structural mapping of the human SAN has revealed that it can be distinguished into head, center, and tail compartments. The head is usually closer to the epicardium, and the tail is typically tilted toward the endocardium [15,16,17,18,19]. Different regions of the SAN pacemaker clusters exhibit diverse functional and molecular features, which are activated through distinct cellular mechanisms [20,21,22] (Figure 1A).

In mice, the SAN artery, autonomic nerve fibers, telocytes, monocytes, macrophages, adipocytes, and fibroblasts were observed via electron microscopy [23]. High populations of fibroblasts in the SAN maintain the source–sink balance and ensure normal electrical conduction [3]. The heterogeneous structure of the SAN makes studying its function challenging. Development of the scRNA-seq technology has aided in examining these heterogeneous and rare cells.

### 2.1. Three-Dimensional Structure of the SAN

In adult mice, the SAN head/center, which is densely packed with clusters of PCs, is the leading pacemaker region, extends superiorly, and wraps the right SVC [24]. The SAN also extends inferiorly toward the inferior vena cava (IVC), forming the tail [25,26]. In mice, the SAN can be genetically divided into a *TBX18^+^/NKX2-5^−^* head domain and a *TBX18^−^/NKX2-5^+^* tail domain [7] (Figure 1B,C). *TBX18* deletion in the SAN head of mice has been shown to result in a normal heart rate [7], whereas *SHOX2* ablation in the SAN tail has led to severe SSS [27]. These findings highlight the different roles of different parts of the SAN in pacemaking and conduction.

Understanding the heterogeneity of the SAN complex is necessary to fully characterize its pacing and conduction functions. To further explore the complex 3D anatomy of the SAN, Goodyer et al. used scRNA-seq in E16.5 mice [10]. The cells underwent unsupervised clustering via t-distributed stochastic neighbor embedding (t-SNE). Cluster 9 showed significant enrichment of nodal markers. Further experiments identified two distinct subclusters within cluster 9, i.e., compact SAN (cSAN) and transitional cells (Tz). cSAN showed high levels of established SAN markers with low or no known atrial myocardium (AM) marker expression [10]. Further investigation of the cellular heterogeneity of the cSAN by Goodyer et al. revealed two distinct clusters in the cSAN, consistent with the previously recognized head and tail subdomains [10]. Both clusters showed high expressions of the nodal markers *HCN4* and *SHOX2*. The head cluster showed increased expression of *TBX18* and decreased expression of *NKX2-5*, whereas the tail cluster showed decreased expression of *TBX18* and increased expression of *NKX2-5* [7], consistent with previous reports [28]. A comparison of these two clusters revealed differentially expressed genes not previously reported. *IGFBP5, PDE1A*, and *VSNL1* were abundant in the head, whereas *SMPX, ALDH1B1,* and *SLC22A1* were abundant in the tail. These findings may enable the examination of different functional characteristics of the head and tail [10].

To further explore the different functional and molecular bases of the SAN head and tail, Li et al. (2019) examined the action potentials (APs) of the SAN’s head and tail separately using a patch clamp and found that the SAN tail has unique APs that mediate the SAN head and A. By performing scRNA-seq assays on SAN regions highlighted by GFP isolated from E13.5 SHOX2^Cre/+^ in R26RmTmG mice, they also verified that NKX2-5 plays an important role in the development and function of the SAN tail. They found four cardiomyocyte subpopulations, one of which expressed both SAN- and AM-specific genes, consistent with the properties of the SAN tail. They also revealed that the SAN tail has unique electrophysiological properties and transcriptomic features [29].

According to Li et al.’s integrated NIOM-3D structural study, the human SAN is functionally insulated/discontinued from the atria by fibrotic tissue, fat, and myofibers, with the exception of 2–5 discrete sinoatrial conduction pathways (SACPs) [19,30]. SACPs transmit electrical impulses to the RA [31,32]. SACP structure includes tracts of myofibers with transitional cells on the SAN border that merge with atrial myobundles [33].

The human fetal SAN contains predominantly PCs and limited collagen and transitional cells. From approximately 2 weeks after birth, Tz gradually increase in number and intertwine with the collagen framework [34]. Importantly, Tz have been implicated in SSS [33]. However, they remain poorly understood due to identification and isolation challenges. Goodyer et al. provided transcriptomic data for Tz [10]. In their study, Tz expressed lower levels of nodal markers and were enriched for AM markers *GJA5* and *SCN5A* [10]. However, no new markers were specific to Tz, and it remains uncertain as to how Tz enter the SAN architecture.

### 2.2. SAN Microenvironment

The unique microenvironment of the SAN maintains its functional characteristics. However, the formation and function of the SAN microenvironment remain poorly understood. To determine the cellular processes that contribute to the formation of the SAN microenvironment, Bressan et al. performed RNA-seq on chicken embryos earlier than E3.5 and found that factors associated with epithelial-to-mesenchymal transition and fibrosis were upregulated in the pacemaker region [35]. Whole-mount in situ hybridization and microsurgery demonstrated that proepicardium-derived mesenchymal cells (including fibroblasts) contributed to the formation of the SAN microenvironment and that the integration of mesenchymal cells is essential to protecting PCs from the electrical “load” of the adjacent AM [35]. However, how mesenchymal cells interact with PCs and regulate physiological functions in the SAN requires further investigation.

Recently, Chou et al. compared *TBX18*-induced PCs and ventricular myocardium via RNA-seq and showed the predominance of glucose metabolism and glycolysis in the GO analysis [36]. *TBX18*-induced PCs co-culturing with fibroblasts activated the integrin-dependent mitogen-activated protein kinase-E2F1 signal through cell–cell contact and induced Aldoc expression in PCs [36]. Their results provided new insights into the link between fibroblasts and PCs and a new way to mediate SSS therapy by regulating Aldoc.

The human SAN comprises 35%–55% fibrotic tissue [37]; cardiac diseases may further increase fibrosis within the SAN, potentially impairing automaticity and the conduction of electricity to the atria. Recently, Li et al. described the transcriptional characteristics of fibroblasts in the SANs of patients with heart failure but did not further elaborate on the connection between fibroblasts and PCs [38].

In addition to fibroblasts, many other cell types exist in the SAN [23]. The interactions among these cells and their roles in maintaining sinus rhythm have not been well elucidated. Single-cell transcriptomics is a promising tool for investigating cell–cell interaction networks [39], which is also the direction of our research group in the future.

## 3. Transcriptional Regulation and Specific Molecular Markers of the SAN

**Figure 2:**
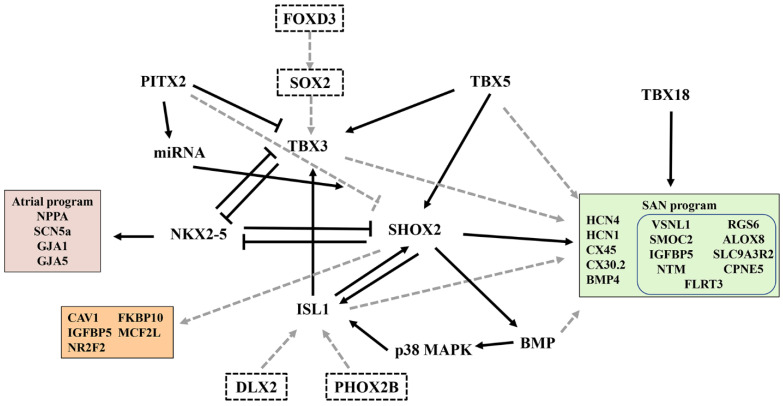
A gene regulatory network that controls SAN development and function. The black arrows indicate gene activation. Black lines with blunt ends represent the inhibition of gene expression by their corresponding transcription factors. The dotted lines indicate indirect or speculation effects. The right box represents the SAN program, and the blue box indicates the SAN-related gene discovered by single-cell sequencing. The left upper box represents the atrial program; the left lower box indicates the genes regulated by *SHOX2*. The dotted box indicates the potential transcription factors associated with the SAN.

SAN formation and function are tightly regulated by a TF network that displays a dynamic and unique expression pattern in the SAN and surrounding AM [40]. These TFs are key players in the development and differentiation of PCs and help maintain pacemaker identity and function. A previous study on SAN formation and development used a gene-deficient mouse model; their findings revealed important aspects of SAN development and differentiation [4,5,6,7,8] (Figure 2). However, how key developmental regulators are regulated in individual cells at specific locations during the SAN development remains unclear.

One of the characteristics of the SAN is the high expression of the hyperpolarization-activated and cyclic-nucleotide-gated ion channel HCN4 [41], which underlies the funny current (If), an essential factor for the maintenance of sinus rhythm [42]. In humans, *HCN4* is expressed in PCs and RA, and so cannot be used for identifying human PCs as the sole marker [43]. CD166/Alcam, a cell surface molecule, can help identify CD166^+^ pacemaker precursors from differentiating mouse embryonic stem cells. However, CD166/Alcam is not expressed in PCs; thus, its application is limited [44,45]. Therefore, specific cell surface molecules of PCs are still being sought.

### 3.1. Transcription Factors

Recently, the critical regulatory role of *ISL1* in SAN development has received increasing attention [46,47]. The specific role of *ISL1* in the SAN is unknown because of the early death of *ISL1*-deficient mice and the loss of cardiomyocytes derived from ISL1 progenitor cells [40]. Recent transcriptome studies have shown significant changes in SAN-related genes when *ISL1* is ablated in the SAN [46]. *ISL1* deficiency in mice leads to the downregulation of *TBX3*, *SHOX2*, and *BMP4*, which are key regulators of SAN development, as well as *HCN4, HCN1*, and *CACNA1G*, which are responsible for the functioning of ion channels of the SAN [46]. However, expressions of *NPPA, PITX2, NKX2.5, GJA1, GJA5,* and *SCN5A* were upregulated. Their study highlighted the central role of *ISL1* in establishing the PC gene program [46].

*SHOX2* plays a key role in PC fate and can activate the pacemaker gene program (*TBX3, ISL1*, and *HCN4*) by repressing *NKX2-5* and the working myocardial gene program [40]. Hoffmann et al. performed transcriptome profiling of SHOX2^+/+^ vs. SHOX2^−/−^ ESC-derived SANLPCs via RNA-seq to explore *SHOX2* pathways involved in pacemaker differentiation [48]. Interestingly, some SAN-related genes (*TBX3, ISL1, HCN4*, and *NKX2-5*) were not affected by *SHOX2* deletion. On combining this finding with data obtained by Vedantham et al. [46], several *SHOX2* target genes were discovered, namely, *CAV1, FKBP10, IGFBP5, MCF2l*, and *NR2F2*, which were validated in mouse and zebrafish models [48]. Their study provided new insights into the transcriptional regulation of *SHOX2* during pacemaker development and function (Figure 2).

The homeobox transcription factor *PITX2* is a laterality gene responsible for establishing the right- and left-body axes, asymmetric gene expression, and organ morphogenesis [49]. Bilateral or ectopic SAN can be found in *PITX2*-deficient embryos and may contribute to atrial fibrillation in adult animals with reduced *PITX2* expression [50,51]. Single-cell transcriptomics has revealed the role of *PITX2* in cardiac development and left–right cellular specification [52]. The CM-RA1 cluster with a SAN transcriptional signature was more abundant in *PITX2* mutants than that in controls [52], highlighting the inhibitory effect of *PITX2* on SAN development.

In addition to the above TFs that affect SAN development and function, next-generation sequencing was recently used to identify other unexplored novel TFs in human SAN and RA and to predict interactions between key TFs and genes involved in pacemaker mechanisms [53]. In the adult SAN, many new TFs were highly expressed (e.g., *FOXD3, DLX2, PHOX2B, VENTX,* and *SOX2*). However, the role of these TFs in the adult mammalian SAN is unknown and should be explored in the future (Figure 2).

A unique set of TFs, which are enriched in PCs, act as activators and repressors and interact with each other to determine the fate of the SAN [40]. Some TFs have been used to direct stem cell differentiation toward SANLPCs [54]; however, the core TF set requires further exploration to obtain highly pure SANLPCs.

### 3.2. Specific Molecular Markers of the SAN

In addition to the enrichment of established nodal genes, scRNA-seq revealed a host of significant novel genes not previously reported to be involved in SAN development or function, including insulin growth factor binding protein 5 (*IGFBP5*) [10], SPARC-related modular calcium-binding protein 2 (*SMOC2*) [10,55], neurotrimin (*NTM*) [10], copine 5 (*CPNE5*) [10], regulator of G-protein signaling type 6 (*RGS6*) [10,56], arachidonate 8-lipoxygenase (*ALOX8*) [55], sodium–hydrogen exchange regulatory cofactor 2 (*SLC9A3R2*) [55], and fibronectin leucine-rich transmembrane protein 3 (*FLRT3*) [57] (Figure 2).

Van Eif et al. used CRISPR/Cas9 to produce SMOC2 frameshift mutation mice and verify the effect of *SMOC2* on SAN function. In vivo and in vitro experiments revealed that *SMOC2* inactivation had little effect on cardiac electrophysiology [55]. Interestingly, *SMOC2* has recently been reported as a new SAN marker. The gene was subsequently validated via immunostaining or fluorescence in situ hybridization, showing *SMOC2* enrichment within the SAN compared with the surrounding AM [10]. In a recent study, scRNA-seq of single cells from the SAN of different mammals identified a species-conserved potential SAN marker, *VSNL1* (a member of the visinin/recoverin subfamily of neuronal calcium sensor proteins), which is abundantly expressed in the SAN but barely expressed in the AM or ventricles [14]. Although its function is unknown, *VSNL1* expression has been detected in the venous pole region of the developing heart [58]. Moreover, *VSNL1* deficiency reduces the heart rate in human-induced pluripotent stem cell-derived cardiomyocytes and mice [14]. *FLRT3*, a cell-autonomous regulator of the adherens junction of PCs, can mediate the transmission of electrical activity by regulating gap junctions [57].

## 4. SAN-Related Signaling Pathways

Studies have elucidated the signaling pathways that affect the development of the atrioventricular and ventricular conduction systems, including the neuregulin/ErbB and endothelin signaling pathways [59]. However, our understanding of the signaling pathways that regulate the proliferation and differentiation of SAN cells and their progenitors remains incomplete [60]. Moreover, the differentiation protocol of human pluripotent stem cells (hPSCs) for generating SANLPCs remains unclear [60]. The molecular and developmental properties of the SAN may contribute to the induction of SANLPCs in vitro. Therefore, transcriptomic analysis of endogenous SAN will enhance the production of SANLPCs efficiently by manipulating the appropriate signaling pathways in vitro.

### 4.1. WNT and BMP Signaling Pathways

According to Vedantham et al. [46], WNT and BMP signaling pathways are enriched in the GO terms associated with SAN-enriched genes at E14.5. Studies have confirmed the effects of both signaling pathways on the SAN. In avian model studies, the right lateral plate mesoderm gives rise to PC progenitors shortly after gastrulation formation in response to WNT signaling cues [61]. Fate-mapping experiments revealed that these progenitor cells migrate to the right inflow region of the heart and differentiate into PCs during embryonic development [61]. Canonical WNT signaling is actively expressed in the SAN and atrioventricular canal regions of E10.5 mouse embryos [62] and can participate in the proliferation of the venous pole TBX18^+^ mesenchymal progenitor cell population [63]. In mouse models, BMP signaling plays a vital role in the specification of mesoderm progenitors in the SAN [64]. In embryonic mice, BMP4 promotes the reprogramming of fibroblasts into cardiomyocytes with spontaneous pacemaker activity [65]. *SHOX2* also plays a key role in SAN development, whereas *BMP4* is a direct target of *SHOX2*, and the expression patterns of *BMP4* and *SHOX2* overlap in embryonic SAN [66]. BMP signaling can prevent ISL1 degradation by phosphorylating *ISL1*, ensuring the transcriptional activity of ISL1 during heart formation [67]. These signaling pathways have been shown to coax hPSCs to the SANLPCs in vitro [68,69].

### 4.2. RA and NOTCH Signaling Pathways

Van Eif et al. [55] performed further studies based on the methods of Vedantham et al. [46] using cell sorting and knock-in TBX3 reporter mice to perform transcriptomic analysis of the SAN. *TBX3* is expressed in the SAN and paces by repressing the expression of working myocardial program genes [5,70]. Through functional annotation analysis of SAN-enriched gene clusters in E17.5, RA and NOTCH signaling pathways were enriched in SAN differential genes, in addition to WNT and BMP signaling pathways mentioned previously [55]. RA, a vitamin A metabolite, promotes the metabolic maturation of embryonic stem cell-derived cardiomyocytes and AM development [71,72]. However, Protze et al. found that RA signaling enhances the pacemaker’s phenotype [69]. NOTCH vitally regulates cell fate determination and cardiogenic differentiation and is essential for ventricular formation and coronary angiogenesis [73]. NOTCH signaling also promotes the abnormal expressions of conduction-related genes, which could direct the differentiation of chamber progenitor cells into conduction-like cells [74]. However, NOTCH induced the electrophysiological transformation of neonatal cardiomyocytes into Purkinje-like ones, rather than SAN phenotypes. Rentschler et al. [75] found that inhibiting NOTCH signaling affects cardiac electrophysiology and leads to atrioventricular node dysplasia, deletion of slow-conducting cells specifically expressing *CX30.2*, and loss of the physiological AV conduction delay. By contrast, recent studies have shown that NOTCH1 deletion in the endocardium leads to defects in the sinus venous valve and SAN development [76]. NOTCH reactivation in vitro leads to a phenotype similar to SSS [77]. Therefore, the effect of NOTCH on the cardiac conduction system requires further exploration.

The signaling clues above were based on successful and effective sampling, and no further screening of the cells was performed, so sample error existed. Goodyer et al. [10] provided unique insights into conduction tissues, including SAN, atrioventricular node/His, and bundle branch/Purkinje fiber, by microdissection of wild-type E16.5 mouse hearts. SAN cells were clustered unsupervised by t-SNE, and cluster 9 showed significant nodal marker enrichment. However, only the BMP and NOTCH signaling pathways were identified in GO terms; the common WNT signaling pathway was not found [10]. Goodyer et al. [10] further validated the previously reported differentially expressed genes in the SAN [46,55]. They found many of these genes to be enriched in other cells, including endothelial, fibroblast, endocardial, and neurons, rather than SAN cells. This may be why scRNA-seq is more efficient than RNA-seq.

**Figure 3:**
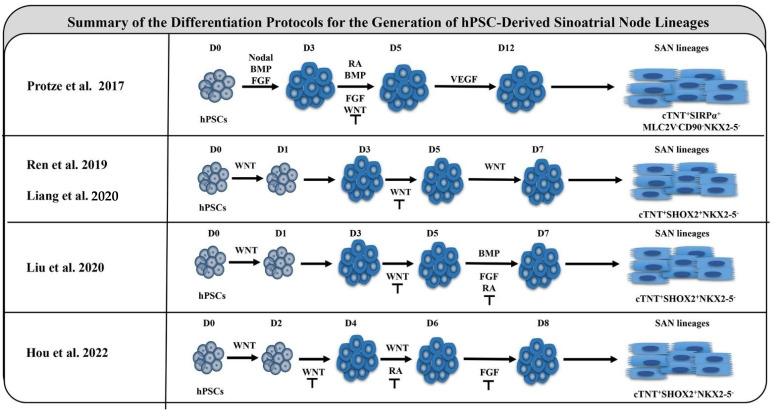
Summary of the differentiation protocols for generating hPSC-derived sinoatrial node lineages. Scheme of the developmentally staged protocol used for sinoatrial node-like pacemaker cells from hPSCs. (Black lines with blunt ends represent the inhibition of the signal pathway; cTNT, cardiomyocyte marker; SIRPAα, pan-cardiomyocyte surface marker; MLC2V, ventricular contractile apparatus protein marker; CD90, mesenchymal marker; NKX2-5, cardiac progenitor marker; SHOX2, conduction cell marker) [68,69,79,82,83].

Although the manipulation of RA signaling alone during hPSC cardiac differentiation does not affect the expression of the cardiac conduction tissue marker *HCN4* [78], in in vitro studies, the combined activation of RA and BMP signaling increases the differentiation ratio of hPSCs to SANLPCs [69] (Figure 3). In zebrafish, a subset of NKX2-5^+^ mesoderm is influenced by canonical WNT5b signaling to initiate the pacemaker program and differentiate into SANLPCs [79]. However, combined manipulation of WNT, BMP, and RA signaling during the myocardial differentiation of hPSCs promotes the development of epicardial lineages, including cardiac fibroblasts and vascular smooth muscle cells [80,81]. By activating BMP or WNT signaling while inhibiting RA and FGF signaling during cardiac differentiation of hPSCs, Liu et al. and Hou et al. significantly promoted the differentiation of hPSCs to SANLPCs [82,83] (Figure 3). Considering the differences in signaling pathway functions, investigators have attempted to suitably regulate signaling pathways to develop more refined protocols for generating highly enriched populations of specific cardiomyocyte subtypes, including atrial, ventricular, and SAN cardiomyocytes [60]. The key signaling pathways that guide the fate decisions between subtypes of cells would benefit from single-cell transcriptomics of the human SAN.

## 5. Physiological Properties of the SAN

Slow diastolic depolarization (SDD) is a unique feature of PCs that drives the generation of spontaneous and rhythmic APs [84]. SDD is subject to autonomic regulation to meet the changing physiological demands in addition to two major cellular mechanisms: the membrane (M-clock) and calcium (Ca^2+^) clocks [85,86]. The unique function of the SAN depends on a complex tissue structure and the specific expression of a range of ion channels, sarcomeric, and gap junction proteins that are expressed in a pattern different from the surrounding AM [8]. Misexpression of these genes leads to electrical remodeling, which is related to the pathogenesis of SSS [87]. However, the regulatory patterns of these key pacemaker channels and associated genes are poorly understood.

### 5.1. Automaticity of the SAN

**Figure 4:**
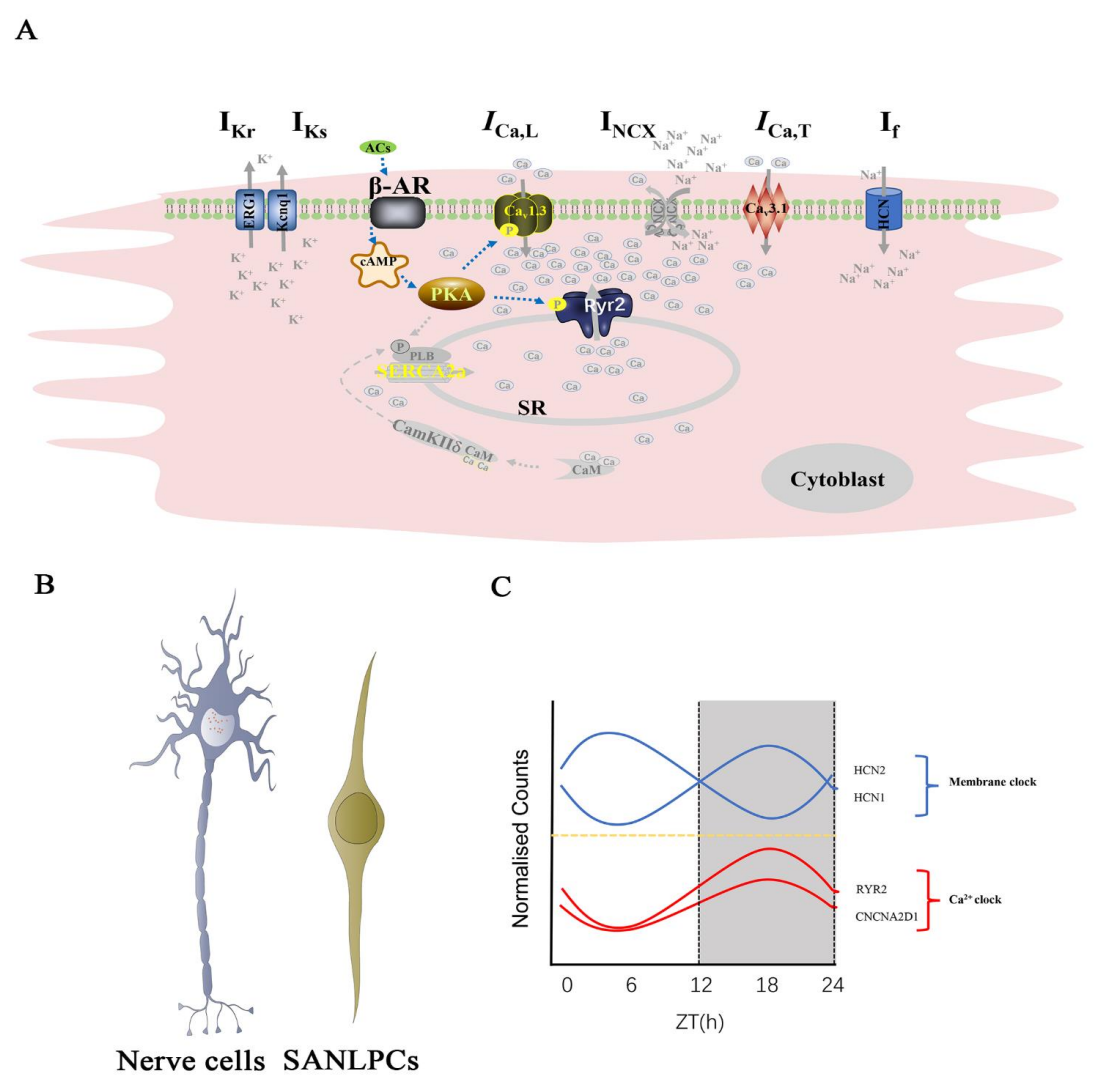
Physiological properties of the sinoatrial node. (**A**) The M-clock includes hyperpolarization-activated cyclic-nucleotide-gated cation channels (I_f_), T-type Ca^2+^ channels (I_Ca,T_), Na^+^/Ca^2+^ exchanger proteins (NCX, I_NCX_), L-type Ca^2+^ channels (I_Ca,L_), and rapid and slow delayed rectifier K+ channels (I_kr_ and I_ks_). The calcium clock includes the sarco-/endoplasmic reticulum Ca^2+^ ATPases (SERCA) modulated by cardiac phospholamban (PLB) and ryanodine receptors (RyR2) located in the sarcoplasmic reticulum membrane. Both the M- and calcium clocks are regulated by the autonomic nervous system through β-adrenergic and muscarinic stimulations, which modulate the activity of protein kinases (cAMP-dependent protein kinase (PKA) and calcium/calmodulin-dependent protein kinase type II (CAMKII)) that phosphorylate multiple proteins in the system. (**B**) Whether the SANLPCs are nerve cells. (**C**) M-clock and Ca^2+^ clock exhibit circadian rhythms.

The molecular mechanisms underlying SAN automaticity are worthy of further investigation. Automaticity is likely generated by either an M- or a Ca^2+^ clock, however, the relative importance of the two is hotly debated [23]. Early theories have suggested that the collection of surface membrane ion channels was sufficient to trigger AP spontaneously, referred to as the M-clock [88]. This concept has driven decades of research on electrical activities and facilitated the identification of numerous ion channels in PCs [89]. The effect of Ca^2+^ on the pacing function was proposed in 1980 [90]. Subsequently, researchers found that the sarcoplasmic reticulum, a major Ca^2+^ store, can spontaneously and rhythmically oscillate Ca^2+^ uptake and release, forming an additional oscillator mechanism in PCs called the Ca^2+^ clock [85]. However, coupled-clock pacemaker systems are now being proposed, in which the M-clock and Ca^2+^ clock synergistically regulate pacing capacity in cardiac PCs [91]. Both mechanisms act interdependently and synergistically to initiate heartbeat [85] (Figure 4A).

Linscheid et al. [23] investigated the protein composition that confers unique properties to the SAN to distinguish the two conflicting interpretations of pacemaking. Although Ca^2+^ clock-related proteins are highly expressed in the SAN, they are also highly expressed in the atria, reflecting the importance of intracellular Ca^2+^ handling for the whole heart. Heterogeneous local calcium signals within and between pacemaker tissue cells are important for synchronizing cardiac impulses [92]. M-clock-related proteins had significantly different expressions, and HCN1 and HCN4 channels were the most favorable for SAN expression [23]. Next, computational modeling was employed to verify the importance of the M-clock. The researchers determined the copy numbers of ion channels per myocyte in the SAN. Their study has important implications for the research on SAN function, as they closely examined SAN pacemaking and the SAN proteome. To date, this is the only proteomic study of the SAN [23].

### 5.2. Neurogenic of the SAN

The SAN is the most densely innervated component of the cardiac conduction system [93]. It is innervated by the autonomic nervous system and regulates the heart rate by modulating ion currents. The very sensitive autonomic responsiveness of the SAN is attributed to its dense distribution of sympathetic and parasympathetic nerves and ganglia [94]. Electron micrographs have also shown autonomic nerve fibers in the SAN [23]. Unsurprisingly, neuronal genes are more abundantly expressed in E17.5 FACS TBX3-Venus^+^ samples [55].

The SAN has an inherent and autonomic rhythm, and its neurons can generate spontaneous electrical impulses [95]. These observations led us to contemplate the SAN–neuron relationship. Chen et al. used single-cell transcriptomic analysis to determine that PCs co-clustered with visual cortex neurons. PCs express cellular markers of glutamatergic neurons containing key elements of the glutamate neurotransmitter system, such as the synthetic glutamate pathway, ionotropic glutamate receptors, metabotropic glutamate receptors, and glutamate transporters. Inhibiting glutamate receptors or transporters reduces the spontaneous pacing frequency of isolated SAN tissues and the spontaneous Ca^2+^ transient frequency in single PCs [96]. In addition, recent studies have shown that voltage-gated sodium channels (Nav) as a major factor in neuronal excitability, play a critical role in pacemaking and conduction [97]. Therefore, we speculate that PCs in SAN, as neuron-like cells, may have more characteristics similar to nerve cells, which need further investigation (Figure 4B).

### 5.3. Circadian Rhythm of the SAN

The circadian system is coordinated by a master clock located in the suprachiasmatic nucleus of the hypothalamus, which directs most of our physiological rhythms [98]. The heart’s electrical activity shows circadian rhythmicity, and rhythm disturbances increase the heart’s susceptibility to arrhythmia [98]. Recently, the denervated SAN exhibited spontaneous beating rates consistent with circadian rhythms and accompanied by the circadian rhythmicity of *HCN4* [99]. However, controlling the circadian rhythm of the SAN requires further investigation.

Wang et al. [100] used RNA-seq to measure the transcriptome and explore the circadian rhythm of the SAN. In contrast to D’Souza [99], who suggested that *HCN4* was responsible for circadian studies, Wang et al. found that *HCN1* and *HCN2* showed distinct circadian rhythms; in contrast, *HCN4* only showed a rhythmic trend. In addition to pacemaker genes that exhibit circadian rhythms, other ion channels, autonomic receptors, downstream pathways, signaling pathways, myofilaments, and metabolism exhibit circadian rhythms. Although both M-clock and Ca^2+^ clock exhibit circadian rhythms, it is not clear if the heart’s circadian rhythm results from post-translational regulation of ion channels by the autonomic nervous system or transcriptional changes (Figure 4C). Given that circadian rhythms are regulated by the nervous system and the SAN contains neural tissues, scRNA-seq might help explore circadian rhythms in the SAN.

## 6. Summary and Future Perspective

SAN is critical for maintaining heart function. However, SAN tissues are heterogeneous and small. Therefore, its molecular biology is challenging. Researchers have attempted to obtain SAN transcriptomic data to gain insights into the molecular mechanisms of the SAN’s regulation and function. RNA-seq was used in the early stages of the study of the SAN transcriptome to generate differential gene expression data from bulk tissues [101]. However, given the heterogeneity and small size of the SAN, separating pure PCs in vivo is challenging. Separating SAN cells inevitably includes neuronal cells, fibroblasts, endothelial cells, and adipocytes. These heterogeneous cellular components interfere with the acquisition of uncontaminated PCs for analysis. In contrast, single-cell transcriptomics can infer the context-dependent phenotype of individual cells and determine the cellular diversity of complex tissues [10,102]. ScRNA-seq has uncovered a host of novel specific markers and unique molecular signatures of various SAN cell subtypes not previously attainable. These provide the foundation for a molecular blueprint of the pacemaker system (Table 1). Spatiotemporal studies of SAN development will expand our understanding of SAN structure and function and provide new ways to understand the pathogenic mechanisms of pacemaker disorders.

SSS may be genetic or secondary to systemic or cardiovascular conditions, but the mechanisms governing SSS are poorly understood because operational cellular or tissue models are either generally deficient or difficult to handle [1,36]. However, the above molecular components critical to SAN function have mostly been studied using animal models, which have functional and anatomical features significantly different from humans. SANLPCs derived from hPSCs may provide a reliable cell source for current studies of human SAN-related diseases. However, the induction protocol of hPSCs inducing SANLPCs requires further improvements. Using the trajectory inference analysis tool URD, we can provide a transcriptional roadmap of PCs and identify the fate decision of hPSCs toward PCs or non-PCs. Therefore, the generation of SANLPCs will benefit from the transcriptome analysis of endogenous SAN.

At present, electronic pacemakers are the main treatment for SSS; however, pacemakers have many disadvantages, such as infection risk, limited battery life, and lack of response to the autonomic nervous system [103]. For these reasons, the development of biological pacemakers is an area of high interest. The ideal biological pacemaker requires PCs embedded in relevant extracellular matrix proteins and surrounded by Tz and other critical cell types to overcome the source–sink mismatch and ensure that autonomously regulated APs propagate to the surrounding myocardial tissues and maintain their rhythm over a long period [104]. SAN transcriptomic data have elucidated the development of biological pacemakers. The discovery of specific signaling pathways and core TFs will improve the generation of SANLPCs in vitro. Recently, Wiesinger et al. have pioneered the application of scRNA-seq to the in vitro differentiation of SANLPC. They have identified the biological functions of different SAN cell types and verified the differences in signaling pathways between different cell types, providing guidance for the targeted differentiation of SAN subtypes in vitro [105]. The revelation of the SAN’s complex anatomy, cellular heterogeneity, and Tz’s transcriptional properties will facilitate the fabrication of biological pacemaker scaffolds and the 3D assembly of PCs. The circadian rhythm and neuroregulation of PCs will help improve the autonomic rhythm of biological pacemakers.

Understanding the SAN characteristics through scRNA-seq tools and techniques has made considerable progress; however, some limitations remain, as follows: (1) isolation of single cells is the first and crucial limiting step in scRNA-seq. Unfortunately, the acquisition of accurate PCs is difficult. (2) The SAN comprises a heterogeneous cell population and functionally similar cell types. We lack specific markers to identify different cell subtypes, and scRNA-seq data cell types should be verified. (3) scRNA-seq breaks down tissues into single cells for study and does not consider the spatial specificity of the cells within the tissue. Because this cell–tissue linkage is important, scRNA-seq data should be biologically interpreted and validated in vivo.

Single-cell technology is a promising approach for exploring cell fate and comprehensively describing the cellular mechanisms conserved in many organisms that control cardiac development. This technology offers a holistic framework for understanding cardiac development. Combining these data with the targeted induction of myocardial differentiation from hPSCs in vitro allows us to translate our knowledge into practical applications and provides a rich resource for understanding the function and structure of the SAN.

**Table 1:** Molecular profiling of the SAN.

References	Experiment Type	Sample Information	New Findings	GEO
Goodyer et al. [10]	scRNA-seq	SAN tissue (Mice)	This study represents the first effort to successfully define the transcriptional profile of the entire murine cardiac conduction system at single-cell resolution.	GSE132658
Liang et al. [14]	scRNA-seq	SAN tissue (Mice, new Zealand white rabbits, and cynomolgus monkeys)	Demonstrated the molecular panorama of SAN cell clusters and the core molecular regulation network underlying the SAN pacemaker activity, and *VSNL1* is a potential SAN marker	
Linscheid et al. [23]	snRNA-seq	SAN tissue (Mice)	Findings from this study present a detailed picture of the SAN by identifying cell type-specific differences in the transcriptome and proteome within the SAN and compared with the surrounding atrial myocardium. Their data support the membrane clock as the key driver of cardiac pacemaking and the first estimate of copy numbers of ion channel subunits, exchangers, and pumps per myocyte in the SAN.	GSE130710
Li et al. [29]	scRNA-seq	SHOX2^Cre/+^; R26R^mTmG^ cells (Mice)	scRNA-seq revealed different transcriptomic characteristics of the SAN head cells and SAN junction cells and verified the role of *NKX2.5* in the development of the SAN	GSE130461
Bressan et al. [35]	RNA-seq	SAN tissue (Chicken)	Proepicardium integrate with pacemaker myocardium facilitates SAN remodeling and is necessary for sustained electrical signal generation and propagation	GSE112894
Chou et al. [36]	RNA-seq	*TBX18* -induced PCs (Mice)	Fibroblasts induce metabolic reprogramming and activate the PC-specific expression of Aldoc within SANs through integrin-dependent cell contact. This study highlights the importance of the SAN microenvironment	
Vedantham et al. [46]	RNA-seq	HCN4-GFP^+^ cells (Mice)	Identified novel SAN-enriched genes, and *ISL1* is a positive transcriptional regulator of the PC gene expression program	GSE65658
Liang et al. [47]	RNA-seq	HCH4-CreERT2^+^ cells (Mice)	The intersection of ChIP-seq data with RNA-seq data revealed genes that were directly regulated by *ISL1*	
Hoffmann et al. [48]	RNA-seq	CD166^+^ SANLPCs (Mice)	To gain insight into direct downstream targets of *SHOX2* that contribute to conduction traits	GSE165544
Hill et al. [52]	scRNA-seq	Cardiac tissue (Mice)	Single-cell transcriptomics to inspect *PITX2* function in cardiac development, cell fates of SHF progenitors, left–right cellular specification, and development of the SAN.	GSE131181
Aminu et al. [53]	RNA-seq	SAN tissue (Human)	The authors determined the key TFs and cell markers with key miRs in the adult human SAN vs. RA tissue	
van Eif et al. [55]	RNA-seq	*TBX3*-Venus^+^ cells (Mice); SAN tissue (Human)	Transcriptome analysis of the SAN reveals new pacemaker markers and signaling pathways but existent contamination from non-PCs	GSE125932
Liang et al. [96]	scRNA-seq	SAN tissue (Mice)	PCs exhibit glutamatergic neuron-like properties	SRP192665
Wang et al. [100]	RNA-seq	SAN tissue (Mice)	Day–night rhythm in the transcriptome of the PCs	
Wiesinger et al. [105]	scRNA-seq	SANLPCS (Human)	This study further diversified pacemaker cardiomyocytes into the “transitional”, “tail”, and “head” subtypes by scRNA-seq, and verified the differences in signaling pathways among these subtypes	GSE189782

## Data Availability

Not applicable.
